# An Angle Estimation Approach for Coherent FDA Radar Based on Transmit-Receive Sum and Difference Beamforming

**DOI:** 10.3390/s26020487

**Published:** 2026-01-12

**Authors:** Jun Zhang, Jingwei Xu, Guisheng Liao

**Affiliations:** 1 National Key Laboratory of Radar Signal Processing, Xidian University, Xi’an 710071, China; 21023110391@stu.xidian.edu.cn (J.Z.); gsliao@xidian.edu.cn (G.L.); 2 Digital Engineering Department, Xi’an Institute of Electronic Engineering, Xi’an 710100, China

**Keywords:** coherent FDA, range-angle two-dimensional coupling, multi-beam transmission, sum and difference beams, target angle estimation

## Abstract

This paper proposes a high-precision angle estimation method based on transmit sum and difference beamforming for coherent frequency diverse array (FDA) radar. By employing a small frequency offset across the array aperture, the coherent FDA radar achieves a range-angle-coupled transmit beampattern that combines wide transmission coverage with narrow reception capability. The proposed method constructs an equivalent two-dimensional coupled sum-difference beam in the target output channel by simultaneously utilizing signal detection outputs from multiple transmitted beams. This approach maintains the inherent advantages of FDA systems while enabling accurate angle estimation without sacrificing coverage. Simulation results demonstrate that the proposed architecture achieves an angular resolution of 1/20 of the beamwidth at a signal-to-noise ratio (SNR) of 20 dB, significantly outperforming conventional techniques. The method exhibits robust performance in various scenarios, which makes it a good candidate for modern radar applications requiring both wide-area surveillance and high-precision angle measurement.

## 1. Introduction

The frequency diverse array (FDA) radar has attracted significant research interests due to its superior capabilities in countering sophisticated electronic jamming and suppressing clutter. In the contemporary complex electromagnetic environment, the demand for high-precision target localization is increasingly critical. Consequently, the application potential of FDA radar in fields such as air defense, missile defense, and precision guidance is substantially pronounced. Advancing the theoretical foundations for angle estimation and elucidating the underlying anti-jamming mechanisms are, therefore, pivotal for enhancing radar system performance. Such research are not only imperative but also hold considerable theoretical and practical merits [1,2]. The fundamental concept of the FDA, which involves applying a small, progressive frequency offset across its radiating elements, enables a unique coupling between the spatial and frequency domains [3]. This paradigm distinguishes it fundamentally from conventional phased array (PA). The mathematical formulas of FDA beamforming have been rigorously derived, and the inherent range-angle coupling characteristic of FDA has been analyzed in detail. By strategically designing the frequency offset, FDA facilitates the generation of a two-dimensional beampattern that exhibits simultaneous dependence on both range and angle dimensions [4,5].

Traditional angle estimation in PA radar is predominantly accomplished through single-pulse method, where digital beamforming (DBF) is implemented and difference beampatterns are formed through channel weighting [6]. To enhance the accuracy of angle estimation, a subarray partition-based approach via local array element processing is proposed in [7], which lacks sufficient robustness in complex electromagnetic environments. As mentioned above, FDA has the potential to realize a higher angle estimation accuracy since it exhibits a range-angle coupling characteristic. In [8,9], the concept of simultaneous angle-dependent matched filtering (ADMF) for coherent FDA radar is elaborated, where the complete time-bandwidth product of the linear frequency modulation (LFM) waveform is leveraged. Through this ADMF method, the range and angle information can be effectively separated by coherent processing. To address practical implementation challenges, phase noise, and system errors in FDA radar are compensated in [10] through the design of an equivalent matched filtering function in the receive dimension and spatiotemporal coding accumulation schemes, thus improving the output signal-to-noise ratio (SNR). For the target detection under low-SNR condition, beamforming and low-sidelobe pulse compression algorithms are often adopted [11,12]. The multiple signal classification (MUSIC) algorithm is introduced into FDA-MIMO radar in [13], where the angular estimation accuracy is analyzed through the Cramér–Rao Lower Bound (CRLB). The challenge of multi-scale moving target detection in non-stationary clutter is addressed in [14], where transmit-dimensional spatiotemporal snapshot training samples are constructed, and spatiotemporal adaptive processing (STAP) is applied in both the receive and Doppler domains, thereby effectively mitigating the issue of insufficient training samples. Joint range-angle estimation under Gaussian noise condition is investigated in [15,16,17,18], where the generalized likelihood ratio test (GLRT) criterion and the maximum likelihood criterion are designed. In particular, random logarithmic frequency offsets are utilized to resolve range ambiguity, and an enhanced subspace-based algorithm is proposed in [15]. From a system-level perspective, a joint design method for wavelength diversity and array geometry is proposed in [19,20,21,22,23,24,25] to mitigate the range-Doppler-angle coupling problem [26,27,28,29,30] and address the degradation in angle estimation accuracy resulting from the inherent range-angle coupling in FDA beams. By correcting errors in the equivalent matched filtering function at the receiver and employing coding techniques to extend the integration time on targets, a high angle estimation precision can be achieved. The transmit array is divided into multiple sub-arrays in [31], where minor frequency offsets are introduced between them. Through the optimization of transmit-side system parameters, joint range-angle estimation is achieved. The coupling between target range and direction of departure (DOD) is addressed in [32] by leveraging the common angular information shared between the multiple input multiple output (MIMO) and FDA-MIMO covariance matrices. An error propagation function is developed in [33] to quantitatively analyze how errors in direction of arrival (DOA) and range estimation affect the accuracy of the estimated DOD and range, thereby enabling precise multi-dimensional parameter estimation in bistatic FDA-MIMO radar systems.

To address the requirement for high-accuracy angle estimation of coherent FDA radar in complex electromagnetic environment, this paper presents a monopulse angle estimation approach by leveraging the transmit degrees of freedom (DOFs) offered by FDA and the time-bandwidth product property of LFM signals. This method employs the synchronous design of LFM baseband waveform with the transmit sum and difference beams. A well-defined spectral notch is systematically generated within the mainlobe region by fixing the direction of the transmit sum beam and designing a difference channel matching filter. This framework preserves the intrinsic range-angle coupling controllability of FDA system while simultaneously achieving significant output SNR enhancement through optimized matched filtering in both sum and difference channels. Comprehensive performance evaluation demonstrates that the proposed framework achieves substantial improvement in angular estimation accuracy within a monopulse duration, outperforming conventional techniques. Meanwhile, the robustness of the method has been verified through extensive simulations under diverse operational conditions, confirming consistent performance across varying SNR scenarios and establishing its practical viability for modern radar applications.

## 2. Signal Model of Coherent FDA Radar

**Figure 1:**
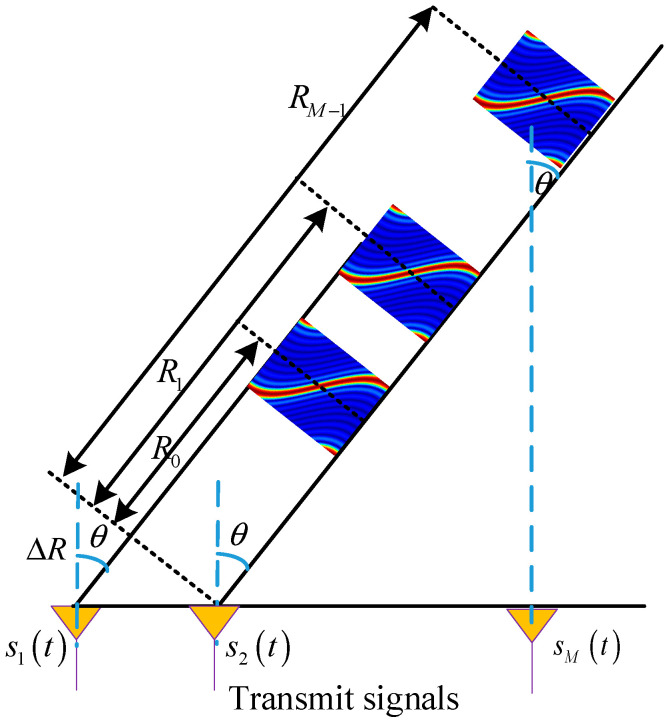
Transmit configuration of coherent FDA.

Consider a uniform linear array (ULA) consisting of *M* elements in FDA radar. The carrier frequency of the signal transmitted by the *m*-th element is $f_{m}=f_{0}+(m-1)\Delta f$ as depicted in Figure 1, where $f_{0}$ represents the carrier frequency and $\Delta f\ll f_{0}$ is the frequency offset of adjacent elements.

For a target with a range of $R_{0}$ and an angle of $\theta_{0}$, $R_{m-1}=R_{0}-(m-1)d\sin\theta_{0}$, *m* = 1, 2, …, *M*, denotes the distance traveled by the waveform emitted from the *m*-th transmitting array element to the target upon its initial propagation, then the propagation delay from the *m*-th array element to the target can be calculated as $\tau_{m}=R_{m-1}/c=\tau_{0}-(m-1)d\sin\theta_{0}/c$ where *d* denotes the inter-element spacing, $\tau_{0}=R_{0}/c$ is the time delay of electromagnetic wave transmitted by the first element to the target with *c* being the operational speed of light. Therefore, the phase of the signal transmitted by the *m*-th element can be expressed as:

(1) $$\phi_{m}(t)=2\pi f_{m}(t-\tau_{m})=2\pi \left[f_{0}+\left(m-1\right)\Delta f\right]\left[t-\frac{R_{0}-(m-1)d\sin\theta_{0}}{c}\right].$$

Further, we organize the formula and its phase terms

(2) $$\phi_{m}(t)=2\pi f_{0}(t-\frac{R_{0}}{c})+2\pi \left(m-1\right)\left(\Delta ft-\frac{\Delta fR_{0}}{c}+\frac{d\sin\theta_{0}}{\lambda_{0}}\right)+2\pi {\left(m-1\right)}^{2}\frac{\Delta fd\sin\theta_{0}}{c}$$

where $\lambda_{0}=c/f_{0}$. The third term can be neglected as it is significantly smaller than the second term in (2). Ignoring the third term $2\pi {\left(m-1\right)}^{2}\Delta fd\sin\theta_{0}/c$, the carrier component $2\pi f_{0}(t-R_{0}/c)$ generates a plane wave phase that varies with respect to $R_{0}$. The second term in (2) consists of three components: (1) Δft, time-dependent scanning term that causes beam direction variation over time *t*; (2) $\Delta fR_{0}/c$, range-dependent coupling term that makes the beam direction dependent on the target range $R_{0}$; and (3) $d\sin\theta_{0}/\lambda_{0}$, spatial angle term that makes the beam direction dependent on angle $\theta_{0}$, which is the same as that of the traditional PA. Therefore, the radar received echo signal can be expressed as

(3) $$\begin{array}{ll} x\left(t,\theta_{0}\right) & =\xi_{0}\sum_{m=1}^{M}\begin{array}{l} \exp\left\{j\pi \mu {\left(t-\tau_{0}\right)}^{2}\right\}\exp\left\{j2\pi f_{d}t\right\}\\ \exp\left\{j2\pi \left(f_{0}+\left(m-1\right)\Delta f\right)(t-\tau_{0}+\left(m-1\right)\frac{d}{c}sin\theta_{0})\right\} \end{array}+n(t)\\ & \approx \xi_{0}\exp\left\{-j2\pi f_{0}(t-\tau_{0})\right\}\exp\left\{j\pi \mu {\left(t-\tau_{0}\right)}^{2}\right\}\exp\left\{j2\pi f_{d}t\right\}\\ & \;\times \exp\left\{j\frac{M-1}{2}\varphi \left(\left.t\right|\tau_{0},\theta_{0}\right)\right\}\frac{\sin\left(\frac{M}{2}\varphi \left(\left.t\right|\tau_{0},\theta_{0}\right)\right)}{\sin\left(\frac{1}{2}\varphi \left(\left.t\right|\tau_{0},\theta_{0}\right)\right)}\;\;+n(t) \end{array}$$

where $\xi_{0}$ denotes the complex scattering coefficient of the target, $f_{d}=2v/\lambda$ denotes the Doppler frequency shift with *v* being the radial velocity of the target; $\exp\left\{j\pi \mu {\left(t-\tau_{0}\right)}^{2}\right\}$ represents the base-band LFM waveform with μ denoting the chirp rate, $\varphi \left(\left.t\right|\tau_{0},\theta_{0}\right)=2\pi \left(\Delta f\left(t-\tau_{0}\right)+d\sin\left(\theta_{0}\right)/\lambda_{0}\right)$ is the phase term with $\left(\tau_{0},\theta_{0}\right)$ related to the target parameters. From (3), it can be observed that the first exponential term is caused by the target delay, the second exponential term corresponds to the delayed LFM waveform, the third exponential term is the Doppler frequency shift caused by the target velocity, and the fourth exponential term and the Sinc function term constitute the transmit pattern of the coherent FDA radar, and *n*(t) represents the additive white Gaussian noise (AWGN).

## 3. Angle Estimation Approach for Coherent FDA Radar Based on Transmit-Receive Sum and Difference Beamforming

This section presents an angle estimation method for transmit sum and difference beamforming in coherent FDA radar. By utilizing the two-dimensional spatio-temporal coupling characteristic of the FDA transmit beampattern, an equivalent symmetric sum/difference beam architecture is designed based on the ADMF. In particular, the difference beampattern is constrained within the effective mainlobe illumination region to construct an optimal difference beam null at the target’s location, thereby achieving an equivalent spatial design for the sum and difference beampatterns in the transmit dimension. Meanwhile, a range-angle matched filtering approach is used to design angle-time-dependent sum and difference matched filters, which enables high-accuracy angle estimation capability.

### 3.1. Joint Transmit-Receive Multi-Beam Processing of Coherent FDA Radar

The spatio-temporally coupled transmit beampattern of coherent FDA radar enables wide-area coverage capability. By employing ADMF, echoes across the full spatial domain can be processed simultaneously. For the echo of each equivalent transmit beam, moving target detection (MTD) is applied to identify high-value targets with enhanced resolution and interference suppression. By leveraging the inherent range-angle two-dimensional coupling characteristic of FDA, a receive-dimensional matched filter is designed. The impulse response of this matched filter is given by:

(4) $$h\left(t|\theta \right)=x{\left(-t,\theta \right)}^{*}=\xi_{0}\exp\left\{-j\pi \mu t^{2}\right\}F_{T}^{*}\left(-t,\theta \right)$$

where $F_{T}\left(t,\theta \right)=\exp\left\{j\frac{M-1}{2}\varphi \left(\left.t\right|\tau_{0},\theta_{0}\right)\right\}\frac{\sin\left(\frac{M}{2}\varphi \left(\left.t\right|\tau_{0},\theta_{0}\right)\right)}{\sin\left(\frac{1}{2}\varphi \left(\left.t\right|\tau_{0},\theta_{0}\right)\right)}$ denotes the transmit beampattern of FDA, and the superscript * represents the conjugation operation.

The echo processed by ADMF can be written as

(5) $$y\left(\left.t,\theta_{0}\right|\theta \right)=x\left(t,\theta_{0}\right)⊛h\left(\left.t\right|\theta \right)$$

where ⊛ denotes the convolution operator. The received signal is convolved with the matched filter’s impulse response to achieve optimal matched reception, thereby enhancing the SNR of the target echo.

In practice, considering a monostatic FDA radar configuration where identical antenna arrays are used for both transmission and reception, the multi-channel received signals undergo simultaneous multi-beam processing. Crucially, the pointing directions of the receive multiple beams should be designed to maintain a one-to-one correspondence with those of the transmit beams. This configuration ensures that the beam widths of the transmit and receive patterns are nearly identical within the ADMF architecture, leading to coherent beam alignment and improved spatial resolution. Without loss of generality, an equivalent transmit-dimensional matched beam can be constructed for any given spatial angle via the ADMF methodology. Subsequent detection and angle estimation processing applied to these beams enable the accurate determination of the target’s angle within the mainlobe region.

### 3.2. Receive Sum and Difference Beam Design for Coherent FDA Radar

In the received signal processing chain, the echo from each channel undergoes a series of operations, including low-noise amplification (LNA), filtering, intermediate frequency sampling, and mixing for down-conversion, and ultimately (3) forms the baseband signal, i.e.,

(6) $$\begin{array}{l} x\left(t,\theta_{0}\right)=\xi_{0}\exp\left\{-j2\pi f_{0}t\right\}\sum_{m=1}^{M}\exp\left\{j\pi \mu {\left(t-\tau_{0}\right)}^{2}\right\}\exp\left\{j2\pi f_{d}t\right\}\\ \exp\left\{j2\pi \left(f_{0}+\left(m-1\right)\Delta f\right)(t-\tau_{0}+\left(m-1\right)\frac{d}{c}sin\theta_{0})\right\}+n(t)\\ \approx \xi_{0}\exp\left\{-j2\pi f_{0}\tau_{0}\right\}\exp\left\{j\pi \mu {\left(t-\tau_{0}\right)}^{2}\right\}\exp\left\{j2\pi f_{d}t\right\}\\ \times \exp\left\{j\frac{M-1}{2}\varphi \left(\left.t\right|\tau_{0},\theta_{0}\right)\right\}\frac{\sin\left(\frac{M}{2}\varphi \left(\left.t\right|\tau_{0},\theta_{0}\right)\right)}{\sin\left(\frac{1}{2}\varphi \left(\left.t\right|\tau_{0},\theta_{0}\right)\right)}+n(t) \end{array}$$

This process is fundamental to preparing the signals for subsequent beamforming and direction finding. This work considers a colocated ULA for both transmission and reception, where the number of transmit and receive elements is equal (*N* = *M*). The multi-channel received echo of a coherent FDA radar for a target at direction *θ* is modeled as:

(7) $$y\left(t,\theta_{0}\right)=\xi_{0}\sum_{n=1}^{N}w_{R}^{H}a\left(\theta_{0}\right)x\left(t,\theta_{0}\right)$$

where $w_{R}$ represents the received weight vector, the superscript *^H^* denotes the transpose conjugate operation, amd $a\left(\theta_{0}\right)={\left[1,e^{j2\pi d\sin\left(\theta_{0}\right)/\lambda_{0}},\cdots ,e^{j2\pi d\sin\left(\theta_{0}\right)\left(N-1\right)/\lambda_{0}}\right]}^{T}$ is the corresponding receive steering vector with the superscript *^T^* being the transpose operation. $x\left(t,\theta_{0}\right)$ denotes the multi-channel echo snapshot received from a specific direction $\theta_{0}$. Then the receive sum and difference beampatterns of the coherent FDA radar can be respectively established as

(8) $$F_{R\_\Sigma }\left(t,\theta \right)=\sum_{n=1}^{N}w_{R\_\Sigma }^{H}a(\theta_{0})$$

and

(9) $$F_{R\_\Delta }\left(t,\theta \right)=\sum_{n=1}^{N}w_{R\_\Delta }^{H}a\left(\theta_{0}\right)$$

where $w_{R\_\Sigma }={\left[1,\cdots ,1,\;\;\;1,\cdots ,1\right]}^{T}\in {\mathbb{C}}^{2Mx1}$ is the sum beam weight vector, which can be constructed via symmetric summation to form a beampattern steered at the array boresight. The vector $w_{R\_\Delta }={\left[1,\cdots ,1,-1,\cdots ,-1\right]}^{T}\in {\mathbb{C}}^{2Mx1}$ denotes the difference beam weight vector, which can be constructed via symmetric sign reversal, where the number of positive and negative elements is equal, resulting in a pattern with a null at the boresight. This joint framework leverages the DOFs in both the transmit and receive dimensions, significantly improving spatial resolution, output SNR, and overall parameter estimation accuracy.

### 3.3. Transmit Sum and Difference Beam Design for Coherent FDA Radar

This subsection introduces a high-accuracy angle estimation method for coherent FDA radar, which is based on equivalent transmit sum and difference beams. Utilizing the target detection result in the previous subsection, equivalent transmit sum and difference beams are designed. Subsequently, the monopulse amplitude comparison technique is employed to achieve high-accuracy angle estimation for detected targets.

As mentioned in Section 3.1, the FDA transmit beampattern inherently possesses a spatio-temporal coupling property. The existing ADMF framework can simultaneously achieve matched filtering and equivalent transmit beamforming. Design the sum channel matched filter as

(10) $$\begin{array}{ll} h_{\Sigma }\left(t|\theta \right) & \;=\exp\left\{-j\pi \mu t^{2}\right\}F_{\Sigma }^{*}\left(-t,\theta \right)\\ & =\exp\left\{-j\pi \mu t^{2}\right\}\exp\left\{-j\frac{M-1}{2}\varphi \left(-\left.t\right|\tau_{0},\theta_{0}\right)\right\}\frac{\sin\left(\frac{M}{2}\varphi \left(\left.-t\right|\tau_{0},\theta_{0}\right)\right)}{\sin\left(\frac{1}{2}\varphi \left(\left.-t\right|\tau_{0},\theta_{0}\right)\right)} \end{array}$$

where $F_{\Sigma }\left(t,\theta \right)=\sum_{m=1}^{M}w_{T\_\sum }^{H}\exp\left\{j2\pi \left(m-1\right)\varphi \left(\left.t\right|\tau_{0},\theta_{0}\right)\right\}$ denotes the transmit sum pattern for the coherent FDA. The sum beam weight vector $w_{T\_\sum }={\left[1,\cdots ,1,\;\;\;1,\cdots ,1\right]}^{T}\in {\mathbb{C}}^{2Mx1}$ can be synthesized using a symmetric summation method to construct a spatial sum beam weight vector, steering the mainlobe towards the antenna boresight direction. Building upon this, the difference channel matched filter is subsequently constructed.

(11) $$h_{\Delta }\left(t|\theta \right)=\exp\left\{-j\pi \mu t^{2}\right\}F_{\Delta }^{*}\left(-t,\theta \right)$$

where $F_{\Delta }\left(t,\theta \right)=w_{T\_\Delta }^{H}a\left(\left.t\right|\tau_{0},\theta_{0}\right)$ represents the transmit beampattern for the difference beam, $w_{T\_\Delta }$ denotes the spatial difference beam weight vector, and $a\left(\left.t\right|\tau_{0},\theta_{0}\right)={\left[1,\exp\left\{j2\pi \varphi \left(\left.t\right|\tau_{0},\theta_{0}\right)\right\},\cdots ,\exp\left\{j2\pi \left(M-1\right)\varphi \left(\left.t\right|\tau_{0},\theta_{0}\right)\right\}\right]}^{T}$ is the transmit steering vector of the coherent FDA. When the number of transmit channels is even, a spatial difference beam weight vector can be constructed using a symmetric sign-alternating sequence, expressed as $w_{T\_\Delta }={\left[1,\cdots ,1,-1,\cdots ,-1\right]}^{T}\in {\mathbb{C}}^{2M\;x\;1}$, where the number of elements with a value of +1 equals the number of elements with a value of −1. Consequently, the coherent FDA transmit difference beampattern can be formulated as the Hadamard product of the difference weight vector and the transmit steering vector

(12) $$F_{\Delta }\left(t,\theta \right)=\sum_{m=1}^{M/2}\exp\left\{j2\pi \left(m-1\right)\varphi \left(\left.t\right|\tau_{0},\theta_{0}\right)\right\}-\sum_{m=M/2+1}^{M}\exp\left\{j2\pi \left(m-1\right)\varphi \left(\left.t\right|\tau_{0},\theta_{0}\right)\right\}$$

The spatial difference beam weight vector is typically determined by solving a system of linear constraint equations at multiple points. By applying constraints to the null at the boresight direction and the half-power beamwidth response of the spatial difference beam, the system of equations can be formulated accordingly.

(13) $$\left\{\begin{array}{l} w_{T\_\Delta }^{H}a\left(\theta_{0}-\theta_{3dB}/2\right)=-1\\ w_{T\_\Delta }^{H}a\left(\theta_{0}\right)=0\\ w_{T\_\Delta }^{H}a\left(\theta_{0}+\theta_{3dB}/2\right)=1 \end{array}\right.$$

where $\theta_{3dB}$ denotes the half-power beamwidth.

Consequently, the spatial difference beam weight vector is derived as follows:

(14) $$w_{T\_\Delta }=\;A{\left(A^{H}A\right)}^{-1}f$$

where $f={\left[-1,0,1\right]}^{T}$ denotes the specified response vector for the spatial difference beam, ***A*** is the matrix composed of spatial steering vectors at the constraint points. The monopulse ratio curve, which is fundamental for angle estimation, is defined as the magnitude ratio of the difference beam output to the sum beam output.

(15) $$k\approx \frac{1}{\theta_{3dB}}\frac{w_{T\_\Delta }^{H}a\left(\theta_{0}+\theta_{3dB}/2\right)}{w_{T\_\Delta }^{H}a\left(\theta_{0}+\theta_{3dB}/2\right)}\;\;\;\;\;\;\;\;\;\;\;$$

where $a\left(\theta_{0}\right)$ represents the array steering vector. The difference beam forms a deep null (*k* ≈ 0) at the boresight direction where the sum beam exhibits the mainlobe, while producing a measurable output (*k* > 0) when the target deviates from the boresight. The variation in the value of *k* with the target’s angular deviation illustrates the effective angular sensitivity established between the sum and difference beams. To further enhance the accuracy of angle estimation, especially for fine accuracy at small angles, several optimization strategies can be implemented. These include refining the beamforming process to deepen the null of the difference beam, adjusting the frequency increment step across the array elements, and optimizing the weight vectors for both the sum and difference beams. Collectively, these measures aim to increase the monopulse ratio slope, thereby improving sensitivity.

Consequently, the transmit-dimensional sum and difference beampatterns for the FDA radar have been successfully derived. This design is adaptable to various beam weight vectors for an arbitrary number of array elements. A fundamental trade-off has been observed between the null depth and the 3 dB beamwidth of the difference pattern. Specifically, a wider 3 dB beamwidth generally correlates with a lower null depth at the null position. By employing the ADMF for sum and difference processing, high-accuracy angle estimation capability can be achieved. Furthermore, when integrated with DBF on reception, this framework facilitates simultaneous multi-beam operation, enabling wide-area coverage with a broad angular scope.

## 4. Numerical Experiments

Numerical experiments are conducted in this section to validate the effectiveness of the proposed transmit-dimensional sum-and-difference monopulse angle estimation method for coherent FDA radar. The system parameters used in the simulations are summarized in Table 1. A scenario with a single target located within the observation sector is taken as an illustration. The specific parameters characterizing the target are provided in Table 2. It is important to note that the SNR in the simulations refers to the input SNR prior to processing by the ADMF and the subsequent receive DBF stages; in other words, it is the SNR of the raw baseband echo signal containing the target. This definition ensures a clear assessment of the processing gain achieved by the proposed method.

### 4.1. Simulation Experiments of Joint Transmit-Receive Sum and Difference Beamforming

**Figure 2:**
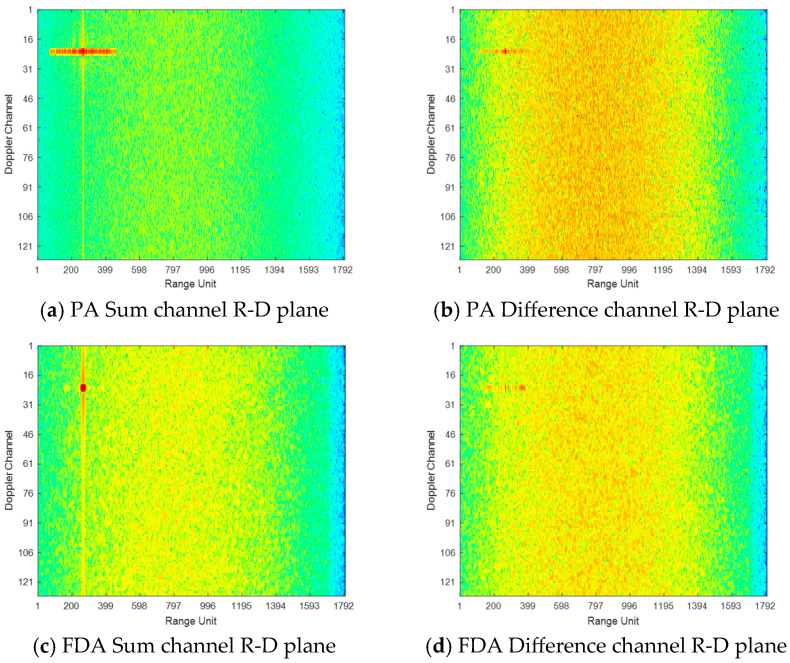
Moving target detection and CFAR results.

This subsection presents simulation results from various stages of the signal processing chain to validate the effectiveness of the proposed processing method for transmit-dimensional sum and difference beams. The multi-channel echoes are firstly subjected to receive digital beamforming (Re-DBF), followed by sequential ADMF, MTD, constant false alarm rate (CFAR) detection, and finally, angle estimation processing. The multi-channel echoes are initially processed by Re-DBF. Operating within the traditional PA monopulse framework, Re-DBF achieves spatial focusing of the target signal through spatial filtering, enabling the precise formation of the sum and difference beams. Figure 2 displays the outputs of PA and coherent FDA radars after MTD processing, where the target detection results are clearly observable. Comparing Figure 2c with Figure 2a, it can be seen that the target detected by coherent FDA exhibits a narrow, focused response in the range dimension, demonstrating superior range resolution compared to traditional PA radar. The significant sharpening of the target in the range dimension, as depicted in Figure 2c, indicates that ADMF, through frequency-domain manipulation, overcomes the limitation of traditional PA radar range resolution being solely determined by the instantaneous bandwidth. Based on the simulation parameters, the processing gains for the Re-DBF, ADMF, and MTD stages can be calculated as 9 dB, 14 dB, and 21 dB, respectively, resulting in a total signal processing gain of 44 dB in coherent FDA radar. Analysis indicates that the output SNR for target is approximately 24 dB, which is in consistence with the theoretical prediction. After CFAR processing, the target is effectively detected. The joint design of the transmit and receive processors effectively reduces the sidelobe levels, particularly mitigating the high sidelobes associated with the joint transmit-receive beampattern. This aids in reducing energy leakage from large targets, thereby diminishing their masking effect on smaller targets. Future work will involve research into low-sidelobe beam design within the joint transmit-receive domain. This method effectively combines the frequency diversity advantage of FDA with the traditional PA signal processing workflow, achieving performance breakthrough offered by the new architecture while preserving the robustness for engineering development.

### 4.2. Simulation of Transmit-Dimensional Difference Beampattern

**Figure 3:**
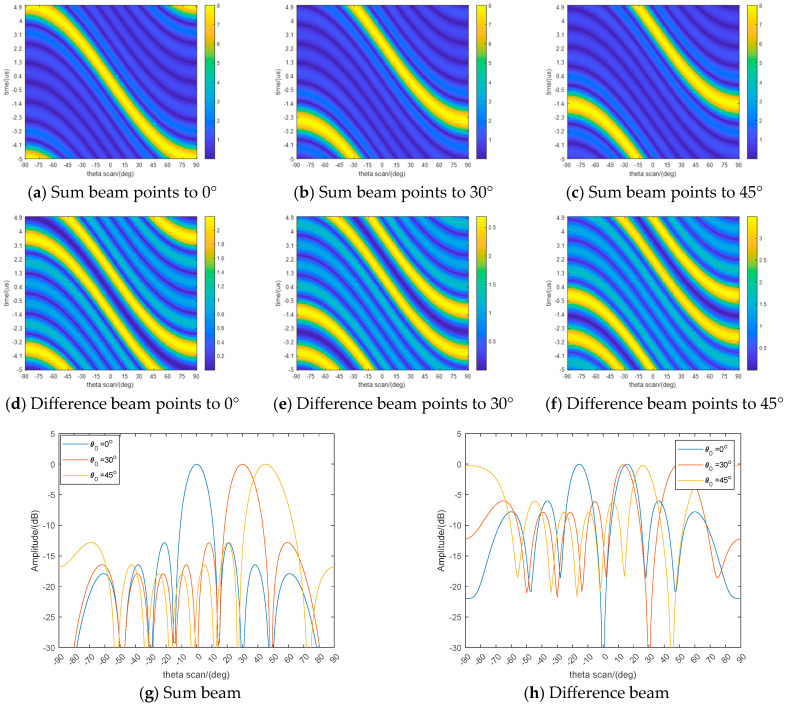
The spatial directions of coherent FDA transmit sum and difference beampattern.

Figure 3 displays the simulation results of the transmit sum and difference beampatterns for the coherent FDA radar. The respective beampatterns and their cross-sectional slices are depicted for beam pointing directions of 0°, 30°, and 45°. The results indicate that effective beampatterns can be synthesized in various steering directions through the beam steering adjustment governed by Equation (13). The coherent FDA radar architecture inherently supports wide-area coverage. By utilizing joint transmit-receive DBF processing, it achieves sum and difference monopulse angle estimation across this extensive area. This capability is realized via a rationally designed frequency-diverse beam scanning mechanism, which facilitates the probing of a broad spatial sector (e.g., azimuth angles of ±90°).

A significant observation from the simulations is the beam broadening effect encountered at extremely large scan angles. Specifically, when the beam is directed towards wide-angle regions (i.e., far from the antenna boresight), both the sum and difference beampatterns show considerable widening. This phenomenon results in a corresponding decrease in the monopulse angle estimation accuracy compared to the performance at or near the boresight direction. The inherent range-angle-time-dependent characteristic of the FDA transmit beampattern underpins this performance trade-off between wide angular coverage and estimation accuracy at the edges of the sector.

**Figure 4:**
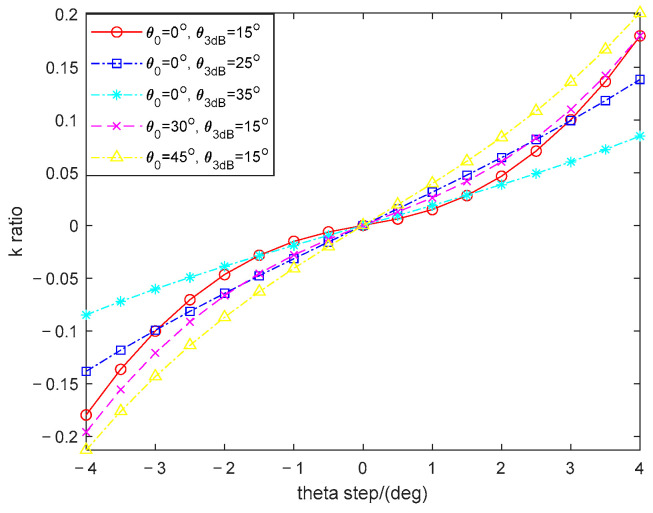
Discrimination curves at different beam directions of coherent FDA radar under 3 dB beam width constraint.

Figure 4 provides the discrimination curves at different beam directions of coherent FDA radar under 3 dB beam width constraint. When the beam is directed to 0°, three half-power beamwidth constraints of 15°, 25°, and 35° are considered, generating three corresponding curves. As depicted in Figure 4, the monopulse discriminanting slopes (*k*-values) for the 15°, 25°, and 35° HPBW constraints at the 0° boresight direction are 10.88, 4.01, and 2.86, respectively. This outcome clearly shows that the monopulse ratio curve associated with the 15° HPBW constraint has a significantly steeper slope. A steeper slope in the monopulse ratio curve directly correlates with higher angular sensitivity, implying that a smaller angular deviation of the target from the boresight results in a larger, more easily measurable change in the difference-to-sum ratio output. As a result, the angle estimation performance is better with the narrower beamwidth. In the sequel, setting the half-power beamwidth constraint as 15°, the beam is directed to 0°, 30°, and 45°, generating another two new curves. It can be clearly observed that degradation in slope at the squint angles of 30° and 45°, which is attributed to the beam broadening effect, a well-known phenomenon in array theory. This performance characteristic aligns with the beam broadening mechanism observed in traditional PA antennas. Consequently, the angle estimation error is significantly higher for targets located in large squint directions compared to those near the boresight. The implications of these findings are particularly significant for low-altitude detection scenarios. In such environments, often characterized by strong ground clutter, precise matching between the sum and difference beampatterns is crucial. Accurate monopulse processing, enabled by a high-gain, narrow sum beam and a well-defined difference pattern with a deep null and a steep slope, facilitates effective discrimination of small, slow-moving, and low-flying targets (often referred to as low-slow-small targets) against background clutter returns.

### 4.3. Analysis of Sum-and-Difference Monopulse Angle Estimation Performance

**Figure 5:**
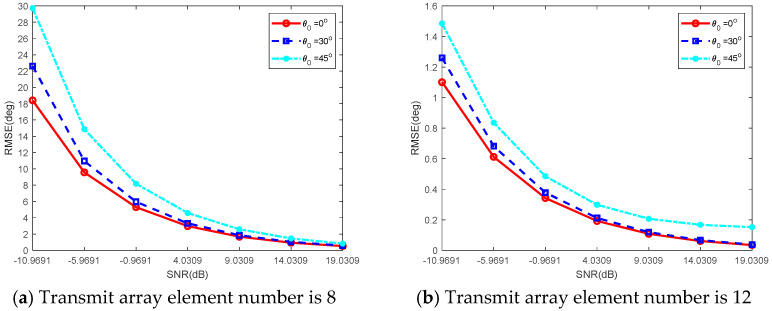
Multi-element angle measurement performance simulation verification.

Figure 5 depicts the performance of angle estimation, quantified by the RMSE, for the coherent FDA radar with respect to the SNR. The simulation results are displayed for two distinct configurations of the transmit array: one with 8 elements and the other with 12 elements. For each configuration, the performance of the ADMF-based sum-and-difference monopulse processing was assessed at beam pointing directions of 0°, 30°, and 45°. As clearly observed in the figure, the angle estimation accuracy improves consistently as the SNR increases. Under high SNR conditions, the RMSE performance curves gradually converge, indicating a regime where the estimation error is dominated by factors other than noise, such as the inherent resolution limitations of the beamforming structure. A key observation is the degradation in performance associated with beam squint angle. The equivalent mainlobe width of the ADMF processing effectively broadens as the beam is steered away from the antenna boresight (0°). Consequently, the angle estimation performance experiences a measurable decrease at the squint angles of 30° and 45° compared to the boresight direction. This phenomenon is attributed to the beam broadening effect. Quantitative analysis at a target output SNR of 20 dB reveals the specific performance metrics. For the 8-element array configuration, the beamwidth is about 15°, and the resulting angle estimation error is approximately 0.75° when the beam is pointed at 0°. In contrast, the 12-element array configuration achieves a narrower beamwidth of approximately 10° and a correspondingly lower estimation error of about 0.45° at the 0° pointing direction. These results demonstrate that the proposed method achieves an angle estimation accuracy of roughly 1/20 of the beamwidth at an SNR of 20 dB. This level of accuracy satisfies the demanding requirements for high-accuracy target angle estimation in practical radar applications, exhibiting the effectiveness of the coherent FDA radar architecture combined with ADMF processing.

## 5. Conclusions

This paper presents a novel transmit-dimensional monopulse angle estimation method for coherent FDA radar. A comprehensive analysis of the equivalent transmit multi-beam signal processing flow is provided, and the equivalence between the constructed transmit-dimensional sum/difference beams and element-domain processing is rigorously derived. The proposed framework possesses the high-accuracy angle estimation capability through transmit sum and difference beamforming. The proposed approach includes two key technical components: transmit sum/difference beamforming and ADMF processing. By employing a spatio-temporal linear multi-constraint approach, the proposed technique enables the design of difference beam for coherent FDA radar with an arbitrary number of array elements. This design flexibility ensures that the constructed sum and difference beams possess adaptable and adjustable characteristics. Simulation results demonstrate that the proposed method achieves an angle estimation accuracy of approximately 1/20 of the beamwidth at a SNR of 20 dB. Furthermore, when integrated with the wide-area coverage capability inherent to ADMF processing, the method facilitates high-accuracy angle estimation for targets across a large angular sector.

## Figures and Tables

**Table 1:** Simulation parameters of coherent FDA radar system.

Parameter	Parameter Values	Parameter	Parameter Values
Carrier Frequency	10 GHz	Frequency Offset	100 kHz
Number Of T/R Array Elements	8/8, 12/12	Element Spacing	0.015 m
Signal Bandwidth	10 MHz	Sampling Rate	40 MHz
PRF	10 kHz	Time Width	10 us

**Table 2:** Simulation parameters of target.

Type	Target Angle	Object Range	Target Speed	SNR	3 dB Beamwidth
Objective	2°	2 km	50 m/s	−20 dB	15° (8 element) 10° (16 element)

## Data Availability

The original contributions presented in this study are included in the article. Further inquiries can be directed to the corresponding author.
